# How to stay perfect: the role of memory and behavioural traits in an experienced problem and a similar problem

**DOI:** 10.1007/s10071-017-1113-7

**Published:** 2017-07-11

**Authors:** Pizza Ka Yee Chow, Stephen E. G. Lea, Natalie Hempel de Ibarra, Théo Robert

**Affiliations:** Centre for Research in Animal Behaviour, Department of Psychology, Washington Singer Building, University of Exeter, Exeter, EX4 4QG UK

**Keywords:** Problem-solving, Generalisation, Positive transfer, Behavioural traits, Memory, Squirrels, Problem-solving efficiency

## Abstract

When animals encounter a task they have solved previously, or the same problem appears in a different apparatus, how does memory, alongside behavioural traits such as persistence, selectivity and flexibility, enhance problem-solving efficiency? We examined this question by first presenting grey squirrels with a puzzle 22 months after their last experience of it (the recall task). Squirrels were then given the same problem presented in a physically different apparatus (the generalisation task) to test whether they would apply the previously learnt tactics to solve the same problem but in a different apparatus. The mean latency to success in the first trial of the recall task was significantly different from the first exposure but not different from the last exposure of the original task, showing retention of the task. A neophobia test in the generalisation task suggested squirrels perceived the different apparatus as a different problem, but they quickly came to apply the same effective tactics as before to solve the task. Greater selectivity (the proportion of effective behaviours) and flexibility (the rate of switching between tactics) both enhanced efficiency in the recall task, but only selectivity enhanced efficiency in the generalisation task. These results support the interaction between memory and behavioural traits in problem-solving, in particular memory of task-specific tactics that could enhance efficiency. Squirrels remembered and emitted task-effective tactics more than ineffective tactics. As a result, they consistently changed from ineffective to effective behaviours after failed attempts at problem-solving.

**Electronic supplementary material:**

The online version of this article (doi:10.1007/s10071-017-1113-7) contains supplementary material, which is available to authorized users.

## Introduction

Problem-solving ability, the ability to overcome obstacles and achieve a goal, has been shown to bring advantages on various measures of fitness. For example, successful problem solvers lay larger clutches of eggs and have increased mating success [review by Boogert et al. (2010), Cauchard et al. (2013), Cole et al. (2012), Keagy et al. (2009), Preiszner et al. (2017); but also see Isden et al. (2013)]. Such impacts on fitness provide a justification for extending investigation to mechanisms that are correlated with problem-solving, such as behavioural traits. However, investigations in such area have only recently begun [Reader and Laland 2003; review by Guez and Griffin (2016)].

An increasing number of studies have now shown that certain behavioural traits are important for problem-solving. The key behavioural traits include persistence, motor diversity (‘behavioural variety’ or ‘exploration diversity’), selectivity (or ‘behavioural selectivity’) and flexibility (e.g. Benson-Amram and Holekamp 2012; Benson-Amram et al. 2013; Biondi et al. 2008; Cauchard et al. 2013; Chow et al. 2016; Diquelou et al. 2016; Griffin et al. 2014; Griffin and Diquelou 2015; Overington et al. 2011; van Horik and Madden 2016; Thornton and Samson 2012). Each of these traits has been shown to relate to problem-solving performance in different ways. For example, increased selectivity enhanced problem-solving efficiency, as measured by decreased latency to solve a problem, in black-throated monitor lizards, *Varanus albigularis albigularis* (Manrod et al. 2008), in Atlantic cod, *Gadus morhua L.* (Millot et al. 2014) and in grey squirrels, *Sciurus carolinensis* (Chow et al. 2016). Increased motor diversity and persistence facilitated success rate in spotted hyenas, *Crocuta crocuta* (Benson-Amram and Holekamp 2012; Benson-Amram et al. 2013) and Indian mynas, *Sturnus tristis* (Griffin et al. 2014), and enhanced problem-solving efficiency in grey squirrels (Chow et al. 2016). Increased flexibility, the rate of switching between tactics, however, decreased solving efficiency in grey squirrels, as a result of decreased selectivity (Chow et al. 2016).

The traits associated with success in a single novel complex task, as discussed above, do not appear to have a fixed hierarchy of importance. A given trait may be salient in relation to a particular task, its context, and perhaps the species involved. For example, selectivity seems to be particularly important when animals return to a learned task after a delay, or experience a new task that resembles one they had experienced previously. With regard to returning to previously experienced task, selectivity appears to be an important factor in the success of captive lions, *Panthera leo,* in solving a suspended puzzle box up to 7 months after experiencing it (Borrego and Dowling 2016), in the success of goats, *Capra hircus,* in solving a two-step food box challenge 10 months after first experiencing it (Briefer et al. 2014), and in the success fat-tailed dunnarts, *Sminthopsis crassicaudata*, when they re-experience a visual reversal learning task (Bonney and Wynne 2002). With regard to situations where animals can apply previously learned tactics in a different (or novel) apparatus through generalisation, selectivity has been shown to be important in stimulus generalisation (e.g. Cuvo 2003), categorisation (e.g. Reichmuth Kastak and Schusterman 2002) and the generalisation of tool use (e.g. Macellini et al. 2012). Such success in transferring previously learnt tactics to a different task depends on individuals being able to recognise that it is the same (or a similar) task, and to recall the tactics that they learned in a previous task.

The ability to recall and employ previously learned tactics to solve a given task after a lapse of time, or to solve a similar task, highlights the interaction between selectivity and cognitive mechanisms such as learning and memory in facilitating problem-solving efficiency. The level of task information retained may affect the way that behavioural traits vary across trials when individuals re-experience the same task. Hypothetical situations include:

1. The ideal outcome, where individuals would immediately perform the effective tactic. In this situation, additional motor diversity (use of alternative tactics), flexibility (switch to another tactic), or persistence (attempts) in solving an experienced problem would not be necessary.
2. The worst-case scenario, where individuals have completely forgotten the task and are learning the task as if at their first experience. In this case, we would expect individuals to increase selectivity (Chow et al. 2016; Manrod et al. 2008; Millot et al. 2014), persistence (Biondi et al. 2008; Chow et al. 2016) and motor diversity (Benson-Amram and Holekamp 2012; Griffin and Diquelou 2015) with increased experience. Flexibility should not vary with increased experience (Chow et al. 2016).
3. The intermediate case, where individuals have retained some but not all information relevant to a previously experienced task. In this case, the variation of traits with trials would depend on how much information they have retained from the past. In this situation, animals may show different types of retrieval strategies. Two strategies have been described: an information-based and a guessing-based strategy. In the information-based strategy, individuals retrieve effective tactics based on the familiarity of the task and retained task information (Malmberg and Xu 2007). Such a strategy implies that there will be switching between retained tactics until asymptotic efficiency is again achieved. In a guessing-based strategy, there will inevitably be errors, but surprisingly it has been shown in humans that these enhance retention, because guessing may lead to more elaborated information processing of correct responses (Yan et al. 2014). Accordingly, if either of these retrieval strategies is used, observed flexibility should increase across trials and should enhance solving efficiency. However, an essential difference between the two strategies lies in the way tactics change as the problem is solved. In the information-based retrieval strategy, changes should not be completely ‘random’ (i.e. behaviours in an individual’s repertoire should not all have equal probability of being exhibited), whereas they should have in the guessing-based strategy.

Here, we examined how memory, alongside behavioural traits, contributes to enhance problem-solving efficiency by giving five grey squirrels, firstly a previously experienced task 22 months after they had last experienced it (hereafter, the ‘recall task’), and secondly a task requiring a previously successful action to be performed in a physically different apparatus (hereafter, the ‘generalisation task’). The squirrels had learned a specific solution for solving a puzzle box involving food reward in the laboratory (hereafter, the ‘original task’) 22 months before the present experiments (Chow et al. 2016). We used Chow and colleagues’ methods to measure four behavioural traits, persistence (rate of attempts), selectivity (proportion of effective behaviours), motor diversity (rate of emitting different types of tactics) and flexibility (rate of switching between tactics after a failed attempt), on a trial-by-trial basis. We chose grey squirrels for this study because they have demonstrated high behavioural flexibility, in a number of situations, including a serial spatial reversal learning task (Chow et al. 2015), a colour reversal learning task (Chow et al. 2017) and a problem-solving task (Chow et al. 2016). Grey squirrels are also known to have good long-term memory, at least in the spatial domain: they are scatter hoarders that cache thousands of nuts during the autumn (Thompson and Thompson 1980), and they are able to relocate their own caches (Jacobs and Liman 1991) and artificial caches (Macdonald 1997) after long intervals of time. While there is always a possibility that memory ability is domain specific, it is reasonable to assume they would be able to remember the solutions to a problem over an extended period. If this is the case, then squirrels would not only be able to solve the task when they re-experience the original task, but they would also show significantly shorter latency to solve the task compared with the first experience of the original task, as in the experiments on lions (Borrego and Dowling 2016) and goats (Briefer et al. 2014) cited above.

We further explored what retrieval strategy squirrels were employing in these two tasks by examining whether squirrels exhibited non-random changes in tactics or not. If squirrels have completely retained the learned task tactics they used to solve the original task, we predict that selectivity would remain at its highest (close to 1 as a proportion), whereas motor diversity, flexibility and persistence would remain at their lowest, and none of these traits would vary with increased experience (see situation 1 above). Such high selectivity would be expected to be one key behavioural trait that enhances efficiency in both tasks. However, as discussed above, if individuals have completely forgotten the task or only retained some information about the original task, then we would observe characteristic variations of these traits with increasing experience in the new situation (see situations 2 and 3 above).

## Methods

### Ethical notes

This study was approved by the Ethical Review Group at the University of Exeter (No. 2012/533), and the experiment was carried out in accordance with the Association for the Study of Animal Behaviour and Animal Behaviour Society guidelines and UK law.

### Subjects and housing

Five squirrels, living in the laboratory, participated in this study. They were named Arnold, Leonard, Sarah, Simon and Suzy and included two females and three males. Their mean age was 6 years; see Supplementary Materials Table S1 for further information on each squirrel. The temperature in their housing was controlled at a constant 19 °C, and lighting was on a 12-h:12-h day–night cycle, with all testing conducted during the light period. The squirrels were housed in large cages that were constructed using metal mesh. In each cage, there was a sliding metal door connected to an overhead tunnel. Only one squirrel was allowed access to the test room at a time for this experiment. A metal mesh divided the test room into two equally large cages (each 1.5 × 1.8 × 2.5 m). The front and ceiling of the cages were metal mesh, whereas the side and the back of the cages were solid concrete wall. One cage had a touch screen panel, set 2 m above the floor as reported in Chow et al. (2017). A camera (Panasonic SWHD-90) was set up in the adjacent cage to capture all behavioural responses during the experiment. Further details of the housing and test room set-up are given by Hopewell et al. (2010). All the squirrels had similar experimental histories in cognitive tasks (see Table S1 in supplementary materials for details). Within the 22 months prior to the present study, the squirrels did not interact with the puzzle box used by Chow et al. (2016) or any similar problem-solving task, nor were they exposed to similar designs as enrichment; they did participate in a serial spatial reversal learning task, as reported by Chow et al. (2015). The squirrels were not food-deprived, and water was provided ad libitum. We ensured squirrels’ motivation by using rewards (hazelnuts) that were different from their daily diet (seeds, fresh fruit and vegetable). Doors allowing the squirrels to enter the test room by the overhead tunnel from their home cages were opened during the times of day when they were most active (0700–1100 and 1500–1800), and tests were carried out when a squirrel entered the test room spontaneously. Data collection took place between May and July 2015.

### Puzzle box for the recall task

**Fig. 1:**
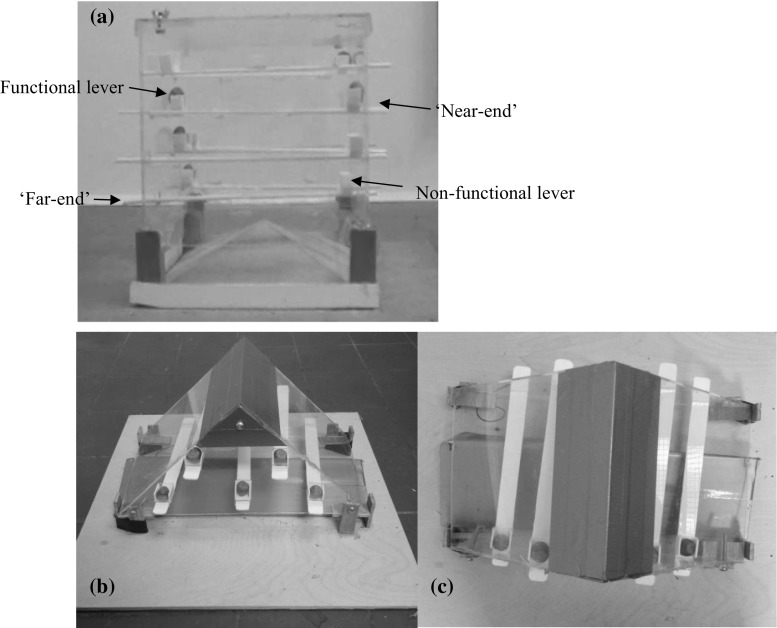
**a** Recall task used the puzzle box that we had presented to the same squirrels 22 months before, as reported in Chow et al. (2016). Puzzle box for generalisation task **b** *front* view and **c** *top* view of the problem apparatus for the generalisation task. This problem is designed to keep the same solution as in the original task, but appears as a novel task for squirrels. A functional lever contains a nut, whereas a non-functional lever is empty. A lever has two ends; the ‘near-end’ refers to the end close to the nut container, whereas ‘far-end’ refers to the end far from the container

Figure 1a shows the puzzle box that was presented to squirrels by Chow et al. (2016), 22 months before the present experiment; we used the same box for this experiment. The box was a transparent Plexiglas cuboidal box (length 25 × width 25 × height 25 cm) that had 10 holes on each side. Ten levers (each lever 29.8 × 1.5 × 0.5 cm thickness), five functional (baited with hazelnuts) and five non-functional (without hazelnuts), were inserted across the box through holes in opposite sides. The holes (2 cm × 0.9 cm W × H) on the box were designed to be larger than the thickness of a lever (0.5 cm), so that squirrels could see and smell the nuts but could not directly reach them after a lever was inserted. At one end of each lever, there was a three-sided container, and this was positioned just inside the box. Four wooden legs were used to support the box, creating a 4.5-cm gap through which squirrels could obtain the hazelnuts once they fell out of the containers. Although squirrels could use many types of behaviours to solve the task, the apparatus was designed so specific behaviours were effective (i.e. the most efficient way) for obtaining a nut and specific other behaviours could not solve the task. The specific effective behaviours were pushing the ‘near-end’ of a lever and pulling the ‘far-end’ (near- and far-end refer to proximity to the hazelnut bait), while the specific ineffective behaviours were pulling the ‘near-end’ of a lever and pushing its ‘far-end’.

### Puzzle box for the generalisation task

Figure 1b, c shows the apparatus used in the generalisation task. It was a transparent puzzle box in the shape of a four-sided triangular prism (triangle front 35 × 19 × 18 cm; length × width × height, rectangular side 25 × 20 cm) with five levers inserted. The puzzle box had completely different physical characteristics and colour than the one used in the recall task, but it still involved moving levers, so that we could examine whether squirrels applied the learned effective and ineffective tactics to obtain the nuts. The length of the levers was shorter than in the recall task, and both ends of each lever were slightly curved (lever dimensions 23.5 × 2 × 0.2 cm L × W × H). The generalisation box had 5 holes (2 × 0.9 cm) on each side, which were horizontally but not vertically aligned with holes on the opposite side. Because squirrels showed a strong preference for choosing the functional levers (with hazelnuts) both in the original (Chow et al. 2016) and in the recall tasks (see results section), we further increased the difference between the recall and generalisation task by including only functional levers. As Fig. 1c shows, both lever ends protruded 1.5 cm out of the box. The box was supported by four wooden legs, creating a 3.5-cm gap from its base. The base of the box (32 × 10 × 3 cm) was a wooden sloped platform (in silver grey colour) which allowed a nut to roll down once it had fallen. As in the recall task, squirrels could see and smell the rewards but could not reach them directly. Squirrels were able to emit the same effective and ineffective behaviours on each lever to obtain a nut: pulling the near-end or pushing the far-end of a lever was ineffective, so they had to push the near-end or pull the far-end.

### Procedures

Squirrels first participated in the recall task, so we could examine whether they remembered the puzzle box they had experienced 22 months ago. The generalisation task was presented 6 days later so as to examine whether squirrels could transfer the same effective behaviours to a physically different box. We kept the same procedures as in Chow et al. (2016) for both the recall and the generalisation tasks; squirrels were tested individually to avoid confounding factors such as stimulus enhancement or social learning in the task. Each squirrel participated in three blocks of four trials in each task (for a total of 12 trials), with a 1-day break between each block (for a total of 14 testing days). In each trial, we placed the box at the centre of the test room. A trial started when squirrels touched or manipulated any part of the box. The trial ended when squirrels completed the task by obtaining all the rewards, when they had not touched the apparatus for 15 min, or when 45 min had elapsed, whichever came first. If a squirrel did not respond, we repeated the trial the next day. This only happened with one squirrel, Suzy, in one trial in the recall task. After every trial, we removed the odour left on the apparatus using disinfectant-impregnated cleaning wipes. We also used wipes after baiting in order to minimise any human scents left on the apparatus. For both tasks, the orientation of the apparatus and the direction the levers faced were pseudo-randomised between trials. For the recall task, we additionally randomised whether a given lever was functional or not. A single success at solving the problem was defined as a squirrel causing a functional lever and/or a nut to drop. A trial therefore normally consisted of five successes.

### Latency measurements

#### Contact latency

For both the recall task and the generalisation task, we measured the latency from when a squirrel entered the test room until it first used its nose or paws to touch the apparatus. We measured the contact latency on the last trial of the recall task and on the first trial of the generalisation task as neophobia. This allowed us to test whether the squirrels perceived the pyramid-shaped apparatus as a novel stimulus in the generalisation task.

#### Success latency

We also measured the time taken to obtain each reward; this was used as a measure of problem-solving efficiency. Latency was timed from the moment when a squirrel started to manipulate a functional lever until the nut it contained dropped. Not every manipulation of a functional lever led to success, but the time spent in unsuccessful manipulation on it was still included. For each trial, we summed all the latencies on functional levers and then divided this total success latency by the number of functional levers that a squirrel solved during that trial, to obtain the mean latency to each success.

### Measurement of behavioural traits

The four behavioural traits, persistence, motor diversity, selectivity and flexibility were measured using methods standardised by Chow et al. (2016). The first author analysed all behaviours from videos using the software Adobe Premiere Pro CS6; this allowed us to analyse behavioural data on a frame-by-frame basis. The behavioural measures of each trait co-vary with one another, and it is therefore necessary to tease them apart analytically to avoid multicollinearity. The measures also need to be normalised in some way, since the longer a trial lasts, the more opportunity there is for a behaviour to be performed. Accordingly, rates of occurrence of behaviours rather than raw counts were used, as in previous experiments (e.g. Biondi et al. 2008; Chow et al. 2016; Griffin et al. 2014; Griffin and Diquelou 2015; Papp et al. 2015). All measurements were taken trial-by-trial for each task (12 trials). For the recall task, we recorded the measures on the functional levers only, to allow direct comparison with the generalisation task in which only functional levers were used.

#### Selectivity

Selectivity was measured as the proportion of effective behaviours. We counted the number of effective behaviours (i.e. either pushing the near-end or pulling the far-end of a functional lever) and the number of ineffective behaviours (i.e. either pushing the far-end or pulling the near-end of a functional lever) in each trial. Then, we divided the number of effective behaviours by the total number of effective and ineffective behaviours for that trial.

#### Persistence

Persistence has been used to assess motivation (e.g. Biondi et al. 2008; Chow et al. 2016; Griffin et al. 2014). We measured persistence as the rate of attempting to solve the problem. An attempt was recorded whenever a squirrel used any of its body parts to manipulate a functional lever, regardless of whether the manipulation was exhibited as effective or ineffective behaviours directed at the box. A new attempt was counted when squirrels switched to a different functional lever or when the squirrel returned to manipulating the same lever after at least one second without having its body in contact with the lever. We counted the total number of attempts in each trial on all functional levers and then divided this number by the total success latency as defined above.

#### Motor diversity

Motor diversity was measured as the rate of using different tactics in solving the problem. We used Chow et al. (2016)’s Table 1 to code the tactics that squirrels used within solving a functional lever. Nine types of behaviour were coded: pull, push in, push up, push down, tilt up, claw, lick, shake and any of two or more of these behaviours occurring simultaneously (combined behaviours). We obtained the rate of motor diversity for each trial by counting the number of types of behaviours that a squirrel exhibited during a trial (ranged from 1 to 9) and then dividing this number by the total success latency for the trial, as defined above.

#### Flexibility

Flexibility was measured as the rate of switching between tactics. A switch was counted whenever a squirrel changed from any of the tactics listed in motor diversity to a different one, regardless of whether either of the tactics involved was effective. We first counted the number of switches between tactics and then divided this number by the total success latency, as defined above, to obtain the rate of flexibility in each trial. To further examine squirrels’ retrieval strategies, we measured the mean number of ‘non-productive’ switches (i.e. switches from effective to ineffective behaviours) across functional levers.

### Data analysis

We used R version 3.3.2 (R Core Team 2016) to analyse all behavioural data. All significance levels reported are two-tailed and were considered as significant when *P* < 0.05.

For the recall task, we used exact binomial tests to examine whether each squirrel was significantly more likely to direct attempts at functional levers (baited with hazelnuts) than at non-functional levers (without hazelnuts). We then pooled the *P* values using Fisher’s formula *χ* ^2^ = −2 Σ In(*P*) (Sokal and Rohlf 1995 p. 794). For the generalisation test, we used a Wilcoxon signed-rank test to assess differences in contact latency from the recall test, and Spearman’s correlation to examine relationships between contact latency and mean success latency on the first trial.

We used generalised estimating equations (GEE) with exchangeable ‘working’ correlation (Hardin and Hilbe 2003) to investigate (1) whether the mean latency to each success in the first trial of the recall task differed from the mean latency to each success in the first trial and the last trial of the original task; (2) whether the mean latency to each success in the last trial of the recall task differed from the mean latency to each success on the first trial of the generalisation task; (3) how the mean latency to each success varied across trials in each task; (4) how each behavioural trait (rate of attempts, rate of flexibility, rate of motor diversity and proportion of effective behaviours) varied across trials; and (5) how the behavioural traits contributed to increasing efficiency in the recall task and in the generalisation task, separately. GEE is a quasiparametric statistical test for model estimates. Because small sample size leads to underestimation of the variance of parameter estimates, we obtained the *P* values using the package ‘geesmv’ (Wang 2015), which adjusted the modified ‘sandwich’ variance estimator (Wang and Long 2011) for estimating the variance–covariance matrix of the parameter estimates. This modified variance has been shown to be robust for experiments that have very small sample size with each individual completing all trials, as in our case.

We used Pearson correlations to explore the relationships between covariates before model testing. Attempt rate and motor diversity were highly correlated in the recall tasks (*r* = 0.78) and in the generalisation task (*r* = 0.86). High correlation was also shown between attempt rate and selectivity (*r* = 0.53) for the generalisation task. To avoid confusion in interpreting the results due to multicollinearity, and, in line with the primary focus of this study on memory for task-effective behaviours, we selected variables for model estimations as follows. We included attempt rate, selectivity, switch rate and trial number for the recall task, but excluded attempt rate was excluded from the model estimation for the generalisation task, because, given the high level of accuracy, it was confounded with the other traits.

## Results

### Performance in the puzzle box recall task

**Fig. 2:**
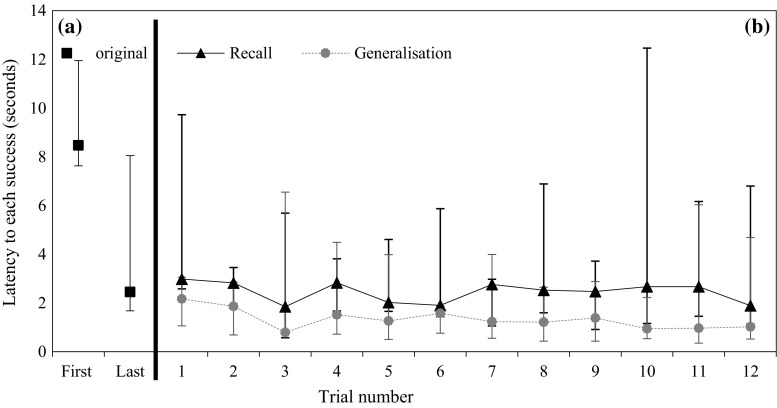
Median, maximum and minimum of mean latency to each success in **a** the first and last trial of puzzle box task. **b** Across 12 trials in the recall tasks, and **c** across 12 trials in the generalisation task. *N* = 5

Figure 2a shows the median across squirrels of mean latency to success in the first trial (8 s) and the last trial (2 s) of the original task. Figure 2b shows the median across squirrels of mean success latency in the first trial of the recall task (3 s). Latency on the first trial of the recall task is significantly different from its value on the first trial of the original task (GEE *χ*_1_^2^ = 4.12, *P* = 0.032), but not different from its value on the last trial of the original task (*χ*_1_^2^ = 2.65, *P* = 0.104). These results indicate some retention for the task 22 months after the last experience with this box. The latency to each success did not vary significantly across trials in the recall task (*χ*_1_^2^ = 0.30, *P* = 0.587).

**Fig. 3:**
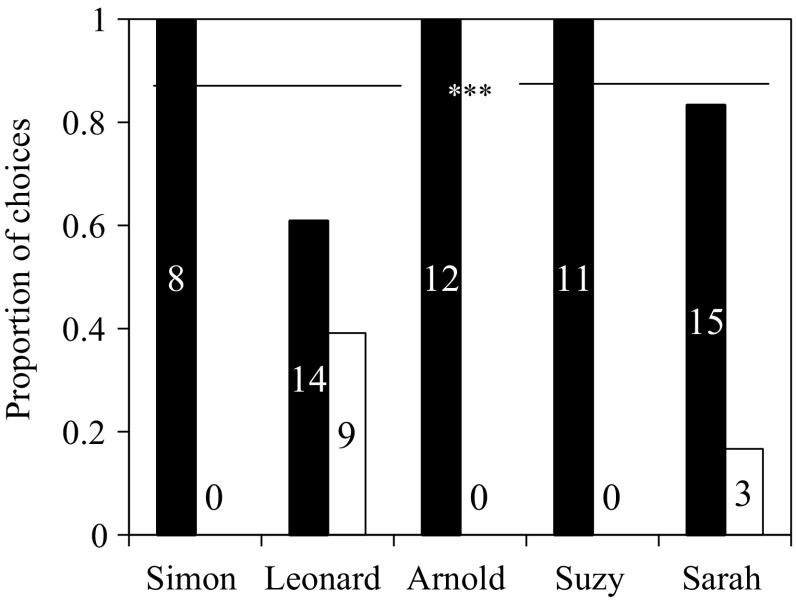
Proportion of attempts between functional levers (■) and non-functional levers (□) in the first trial of recall task.* Number inside each bar* indicates the actual number of attempts. *N* = 5, ***P < 0.001

Figure 3 shows that on the first exposure of the recall task after 22 months, squirrels made more attempts to solve functional levers (with hazelnuts) than to solve non-functional levers (without hazelnuts). This preference for solving functional levers was significantly above chance (pooled binominal tests: *χ*_10_^2^ = 49.25, *P* < 0.001). Although squirrels could smell the nuts, their behaviours suggested that they often first used vision to approach a functional lever before using olfaction, presumably to assess the quality of a nut rather than locating the nut (See supplementary video VS1–S2).

**Fig. 4:**
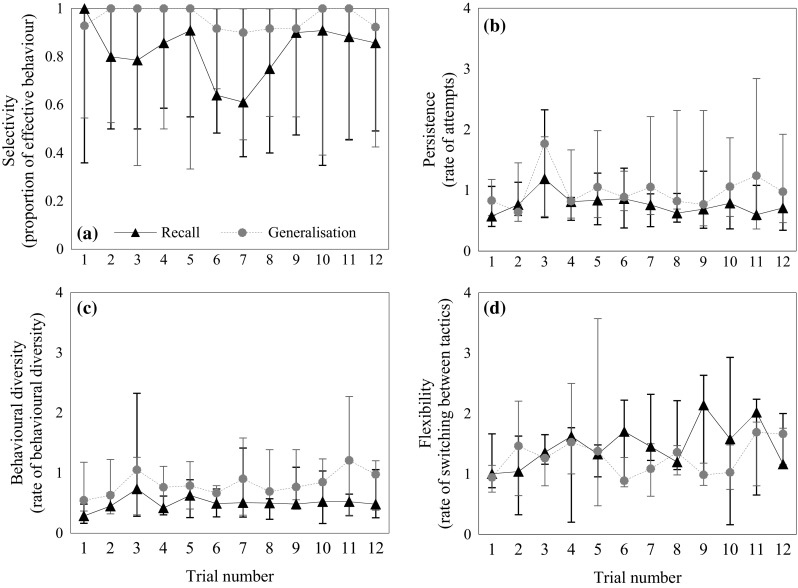
Medians (±minimum and maximum) of each behavioural trait across trials in the recall task and the generalisation task. **a** Behavioural selectivity, the proportion of effective behaviours, across trials. **b** Persistence, the rate of attempts, across trials. **c** Behavioural diversity, the rate of behavioural diversity, across trials. **d** Flexibility, the rate of switching between tactics, across trials. *N* = 5

Figure 4a shows the variation of selectivity (i.e. the proportion of effective behaviours) across trials in the recall task. Selectivity did not vary significantly across trials (*χ*_1_^2^ < 0.001, *P* = 0.95). Figure 4b–d shows the variations of persistence, motor diversity and flexibility, respectively, in the recall task. With increased experience, squirrels significantly increased flexibility (*χ*_1_^2^ = 6.42, *P* = 0.011), but not persistence (*χ*_1_^2^ = 0.05, *P* = 0.826) or motor diversity (*χ*_1_^2^ = 0.67, *P* = 0.414). The median across squirrels of mean number of non-productive switches (i.e. switches from effective to ineffective behaviours) was 0.6 and 0.4 in the first trial and the last trial of the recall task, respectively.

### Performance in the generalisation task

We first verified that squirrels perceived the generalisation puzzle box as a different stimulus. We compared the latency to contact the apparatus in the last trial of the recall task with the first trial of the generalisation task. All squirrels took longer to approach the puzzle box in the first trial of the generalisation task (median of mean latency = 23 s) than in the last trial of the recall task (median of mean latency = 11 s), and this difference was significant (Wilcoxon signed-rank test: W = 5, *Z* = −2.02, *P* = 0.043). Neophobic responses towards the generalisation apparatus was not correlated with the latency to each success in the first trial of the generalisation task (*r*_*s*_ = 0.30, *P* = 0.623).

Figure 2b shows the median of mean latency to each success across 12 trials in the generalisation task. All squirrels took significantly less time to each success in the first trial of the generalisation task (median of mean latency = 2 s) than in the first trial of the recall task (*χ*_1_^2^ = 4.39, *P* = 0.036). However, the latency to each success was not significantly different between the last trial of the recall task (median of mean latency = 1 s) and the first trial of the generalisation task (*χ*_1_^2^ = 0.67, *P* = 0.413). Figure 4a shows that squirrels consistently showed a high proportion of effective behaviours (median proportion across squirrels = 0.93) in the first trial of the generalisation task. This proportion did not vary significantly across trials (*χ*_1_^2^ = 0.38, *P* = 0.536), which suggested they quickly perceived the problem as the same despite the changed appearance of the task. Figure 2b shows that the latency to each success did not significantly vary across trials (*χ*_1_^2^ = 1.61, *P* = 0.205). Figure 4b–d shows the variation of persistence, motor diversity and flexibility during the generalisation task. None of the three behavioural traits varied significantly across trials (persistence: *χ*_1_^2^ = 1.12, *P* = 0.290; motor diversity: *χ*_1_^2^ = 0.54, *P* = 0.461; flexibility: *χ*_1_^2^ = 0.06, *P* = 0.808). The median across squirrels of the mean number of non-productive switches (i.e. switches from effective to ineffective behaviours) was 0.2 in both the first trial and the last trial of the generalisation task.

### Factors associated with problem-solving efficiency in the recall task

Table 1 (left panel) shows the results of the correlational analysis for the recall task. Model 1 shows that only selectivity, measured as the proportion of effective behaviours, and flexibility, measured as the rate of switching to other tactics after a failed attempt, were significantly associated with efficiency. Specifically, high efficiency was related to a high proportion of effective behaviours (*χ*_1_^2^ = 25.39, *P* < 0.001) and a high switch rate (*χ*_1_^2^ = 6.17, *P* = 0.013). As with our previous finding in Chow et al. (2016), the non-significant effects of persistence, measured as the rate of attempts, and experience, recorded as trial number, suggest that selectivity and flexibility mediated the effect of experience and persistence on efficiency. Therefore, we ran two separate analyses to examine two covariates, experience and persistence, in relation to response variable, selectivity, in one model (Model 2) and flexibility in another model (Model 3). Model 2 (left panel) shows that selectivity was related to persistence (*χ*_1_^2^ = 22.65, *P* < 0.001), but not experience (*χ*_1_^2^ < 0.01, *P* = 0.989); higher persistence was associated with higher selectivity. Model 3 shows that flexibility was significantly related to both persistence (*χ*_1_^2^ = 6.03, *P* = 0.014) and experience (*χ*_1_^2^ = 4.85, *P* = 0.028); increased flexibility was positively correlated with higher persistence and increasing experience. We ran a final model (Model 4) to examine whether persistence increased across trials, and results showed it did not (*χ*_1_^2^ = 0.05, *P* = 0.826). These results imply that persistence, a trait that is not affected by experience, affects efficiency on the recall test, but does so indirectly through increasing selectivity and flexibility.

**Table 1:** Summary for GEE models including estimates, Chi-square values (*χ* ^2^), *P* values, effect size of each path (Path *β*), and total effect size (Total *β*) of each covariate

DV	Covariates	Recall test					Generalisation task				
		Estimates	*χ* ^2^	*P*	Path *β*	Total *β*	Estimates	*χ* ^2^	*P*	Path *β*	Total *β*
Model 1											
Latency to each success	Experience	<0.01	<0.01	0.969	0.01	−0.10	−0.02	0.80	0.371	−0.06	−0.11
	Selectivity	−7.69	25.39	**<0.001**	−**0.74**	−0.74	−5.37	12.05	**<0.001**	−**0.88**	−**0.88**
	Persistence	−0.65	0.86	0.353	−0.17	−0.47	**–**	**–**	**–**	**–**	**–**
	Flexibility	−1.04	6.17	**0.013**	−**0.38**	−0.38	−0.06	0.13	0.713	−0.02	−0.03
Model 2											
Selectivity	Experience	<0.01	<0.01	0.989	<0.01		<0.01	0.37	0.544	0.06	
	Persistence	0.10	22.65	**<0.001**	**0.26**		**–**	**–**	**–**	**–**	
	Flexibility	**–**	**–**	**–**	**–**		0.01	0.06	0.812	0.01	
Model 3											
Flexibility	Experience	0.05	4.85	**0.028**	**0.22**		0.01	0.06	0.808	0.05	
	Persistence	0.41	6.03	**0.014**	**0.29**		**–**	**–**	**–**	**–**	
Model 4											
Persistence	Experience	<0.01	0.05	0.826	0.03						

Note that all measures are taken trial-by-trial (12 trials in total). Experience was recorded as trial number (total 12 trials); persistence was measured as the rate of attempts; behavioural selectivity was recorded as the proportion of effective behaviours (i.e. either push the ‘near-end’ or pull the ‘far-end’); flexibility was measured as the rate of switching to another type of tactic after a failed attempt

Bold number indicates *p* < 0.05

### Factors associated with problem-solving efficiency in the generalisation task

Table 1(right panel) shows the results for the generalisation task. Model 1 shows that the latency to each success was significantly related to selectivity (*χ*_1_^2^ = 12.05, *P* < 0.001); a higher proportion of effective behaviours was associated with greater efficiency. Model 2 (right panel) shows that in this task, selectivity was not related to experience (*χ*_1_^2^ = 0.37, *P* = 0.544) or flexibility (*χ*_1_^2^ = 0.06, *P* = 0.812). The final analysis (Model 3) shows that flexibility was not significantly related to experience (*χ*_1_^2^ = 0.06, *P* = 0.808).

### Total effect of behavioural traits on efficiency

For each model, we obtained the effect sizes for each trait (Path *β*). We then calculated the total effect (Total *β*) for each trait in Table 1. For both tasks, selectivity showed the highest effect on efficiency (in the recall task: *β* = −0.74 and in the generalisation task: *β* = −0.88).

## Discussion

In this study, we examined how memory and behavioural traits improved problem-solving efficiency when squirrels re-experienced a task that reappeared after a substantial time had elapsed (the recall task), and when squirrels encountered the same problem in a different apparatus (the generalisation task). We showed that all squirrels retained some information from previous experience by showing a high proportion of effective behaviours (Fig. 4a), indicating that their retention of the task extended to the specific tactics that were effective in solving it (i.e. pushing at the near-end or pulling at the far-end of a lever). Such information about task tactics facilitated squirrels’ solution of the problem. All squirrels also successfully transferred the same tactics to solve the problem when it appeared in a physically different apparatus. Aside from memory, selectivity and flexibility were important factors in increasing efficiency in the recall task, whereas only selectivity affected efficiency in the generalisation task.

As discussed in the introduction, the level of retained task information may affect how traits vary in the recall and the generalisation tasks. In our case, other than flexibility in the recall task, none of the traits varied with increased experience, suggesting that the squirrels remembered the task almost perfectly (Fig. 4a–d). The fact that squirrels consistently showed a high proportion of effective behaviours (Fig. 4a) is in line with our prediction that memory and selectivity are tightly related to each other and their interaction is the key trait to advanced problem-solving efficiency in both tasks (Table 1). In both tasks, memory of which tactics were effective may reflect a series of associations that have been formed in the past; for example, an association between the cues, the behaviours and the rewards formed by operant conditioning would allow the squirrels to promptly locate functional levers (Fig. 3), emit the effective behaviours and obtain the hazelnut.

Another behavioural trait, flexibility, measured as the rate of switching between tactics after a failed attempt, was also found to be an important trait for achieving efficiency in the recall task, but not in the generalisation task (Model 1). Flexibility also varied with increased experience in the recall task (Fig. 4d). In the introduction, we discussed two possible retrieval strategies under a recall situation: individuals would either explore the effective tactics based on retained information (information-based) or explore all possible tactics (guessing-based strategy). In both tasks, squirrels showed more effective behaviours than ineffective behaviours, suggesting they were using an ‘information-based’ strategy so that if they did emit an ineffective behaviour, they quickly switched to an effective one. These results show how flexibility is related to memory, because a productive change of tactics would involve remembering the correct tactic and may also lead to the reinforcement of the effective behaviours during the recall task. It follows that productive changes of tactics allowed the squirrels to achieve efficiency in the recall task and to apply the same tactics from the first trial of the generalisation task (Fig. 4a).

The final trait of particular interest in this study is persistence, measured as the rate of making attempts to solve the problem. The role of persistence in solving novel problems is well established (Benson-Amram and Holekamp 2012; Biondi et al. 2008; Chow et al. 2016; Griffin et al. 2014, Papp et al. 2015, Thornton and Samson 2012; van Horik and Madden 2016). Persistence may largely reflect the motivation of individuals (e.g. Biondi et al. 2008; Chow et al. 2016). When squirrels re-experience the same task, such motivation embraces various aspects of problem-solving, including goal-orientation to food reward (Fig. 3), changes to another tactic after a failed attempt (Model 2 and Model 3) and motivation to emit the effective behaviours to increase problem-solving efficiency (Table 1 Model 2). However, unlike others who have argued that persistence may not be involved in any cognitive process that could lead to problem-solving success (e.g. van Horik and Madden 2016; Thornton and Samson 2012), we suggest that such persistence may well interact with cognitive processes in several ways. For example, paying attention to the functional cues or properties of the task has been demonstrated in kea and crows using tools to solve a problem (e.g. Auersperg et al. 2011; St Clair and Rutz 2013; Werdenich and Huber 2006). In our case, squirrels may pay attention to cues such as the levers that contain hazelnuts to locate which lever to solve. But unlike what has been found in tool-use studies, the use of cues did not develop with increased experience during problem-solving. Squirrels showed an immediate strong preference to contact functional levers rather than non-functional levers, both when they first encountered this puzzle box 22 months prior to this study (Chow et al. 2016) and in the first trial of the recall task (Fig. 3). These results imply that squirrels quickly focused their attention on the reward and reward-related components of the apparatus (levers) from their first encounter with the puzzle box. Such attention may be developed from previous handling or knowledge about the objects or food (e.g. Bird and Emery 2009; Taylor et al. 2010). The effect of persistence was positively mediated by flexibility and selectivity in the recall task (Model 3); that is, persistence was indirectly related to the latency to solve the task through its effect on selectivity and flexibility. Given that persistence was also highly correlated with selectivity in the generalisation task, we suggest that higher motivation led the squirrels to emit effective behaviours in this task. Taking these trends together, one could deduce that persistence is also related to retrieval from memory, which may also explain why persistence did not show a significant increase (or decrease) across trials in either task (Fig. 4b).

The sample size in the present study was limited, and hence, we had limited degrees of freedom available to explore other interactions between traits on problem-solving efficiency, and we do need to be cautious in generalising from five squirrels to the whole species. Nevertheless, we have shown that learning tactics for a given task can improve future problem-solving efficiency if individuals are able to recall these tactics when they revisit the same task, or when it is possible to apply them in a different apparatus. In these situations, learning plays a minimal role, whereas long-term memories of the effective tactics along with other factors are important for increasing efficiency. In a broader context, these results highlight the mechanisms, including cognitive capacity and behavioural traits, that are correlated with problem-solving ability and enable animals to achieve better problem-solving performance. This provides information about why these mechanisms evolved together. In turn, it should be possible to investigate which of these factors are general across a range of tasks, thereby making it plausible to try to obtain a measure of general cognitive ability (‘*g*’) for an individual. It should also be possible to study which of these factors might differ between species—for example, between the Eastern grey squirrel and the Eurasian red squirrel, which it has largely replaced in Britain and Ireland. Ultimately, we would hope to be able to highlight the factors that explain the varied fitness consequences associated with cognitive capacity, for example the relationship between ‘*g*’ and the success of some species as invaders of new environments.

## Electronic supplementary material

Below is the link to the electronic supplementary material.

Supplementary material 1 (DOCX 17 kb)

Supplementary material 2 (MP4 83444 kb)

Supplementary material 3 (MP4 41739 kb)

## References

[CR1] Auersperg AMI, von Bayern AMP, Gajdon GK, Huber L, Kacelnik A (2011). Flexibility in problem solving and tool use of kea and New Caledonian crows in a multi access box paradigm. PLoS ONE.

[CR2] Benson-Amram S, Holekamp KE (2012). Innovative problem solving by wild spotted hyenas. Proc R Soc Lond B.

[CR3] Benson-Amram S, Weldele ML, Holekamp KE (2013). A comparison of innovative problem-solving abilities between wild and captive spotted hyenas (*Crocuta crocuta*). Anim Behav.

[CR4] Biondi LM, Bó MS, Vassallo AI (2008). Experimental assessment of problem solving by *Milvago chimango* (Aves: Falconiformes). J Ethol.

[CR5] Bird CD, Emery NJ (2009). Rooks use stones to raise the water level to reach a floating worm. Curr Biol.

[CR6] Bonney KR, Wynne CD (2002). Visual discrimination learning and strategy behaviour in the fat-tailed dunnart (*Sminthopsis crassicaudata*). J Comp Psychol.

[CR7] Boogert NJ, Fawcett TW, Lefebvre L (2010). Mate choice for cognitive traits: a review of the evidence in nonhuman vertebrates. Behav Ecol.

[CR8] Borrego N, Dowling B (2016). Lions (*Panthera leo*) solve, learn, and remember a novel resource acquisition problem. Anim Cogn.

[CR9] Briefer EF, Haque S, Baciadonna L, McElligott AG (2014) Goats excel at learning and remembering a highly novel cognitive task. Front Zool 11:20. http://www.frontiersinzoology.com/content/11/1/20 10.1186/1742-9994-11-20 PMC3987177 24666734

[CR10] Cauchard L, Boogert NJ, Lefebvre L, Dubois F, Doligez B (2013). Problem-solving performance is correlated with reproductive success in a wild bird population. Anim Behav.

[CR11] Chow PKY, Leaver LA, Wang M, Lea SEG (2015). Serial reversal learning in grey squirrels: learning efficiency as a function of learning and change of tactics. J Exp Psychol Anim Learn Cogn.

[CR12] Chow PKY, Lea SEG, Leaver LA (2016). How practice makes perfect: the role of learning, flexibility, and persistence in problem solving efficiency. Anim Behav.

[CR13] Chow PKY, Leaver LA, Wang M, Lea SEG (2017). Touch screen assays of behavioural flexibility and error characteristics in Eastern grey squirrels (*Sciurus carolinensis*). Anim Cogn.

[CR14] Cole EF, Morand-Ferron J, Hinks AE, Quinn JL (2012). Cognitive ability influences reproductive life history variation in the wild. Curr Biol.

[CR15] Cuvo AJ (2003). On stimulus generalization and stimulus classes. J Behav Educ.

[CR16] Diquelou MC, Griffin AS, Sol D (2016). The role of motor diversity in foraging innovations: a cross-species comparison in urban birds. Behav Ecol.

[CR17] Griffin AS, Diquelou MC (2015). Innovative problem solving in birds: a cross-species comparison of two highly successful passerines. Anim Behav.

[CR18] Griffin AS, Diquelou M, Perea M (2014). Innovative problem solving in birds: a key role of motor diversity. Anim Behav.

[CR19] Guez D, Griffin AS (2016). Unraveling the key to innovative problem solving: a test of learning versus persistence. Behav Ecol.

[CR20] Hardin JW, Hilbe JM (2003). Generalized estimating equations.

[CR21] Hopewell LJ, Leaver LA, Lea SEG, Wills AJ (2010). Grey squirrels (*Sciurus carolinensis*) show a feature-negative effect specific to social learning. Anim Cogn.

[CR22] Isden J, Panayi C, Dingle C, Madden J (2013). Performance in cognitive and problem-solving tasks in male spotted bowerbirds does not correlate with mating success. Anim Behav.

[CR23] Jacobs LF, Liman ER (1991). Grey squirrels remember the locations of buried nuts. Anim Behav.

[CR24] Keagy J, Savard J-F, Borgia G (2009). Male satin bowerbird problem solving ability predicts mating success. Anim Behav.

[CR25] Macdonald IMV (1997). Field experiments on duration and precision of grey and red squirrel spatial memory. Anim Behav.

[CR26] Macellini S, Maranesi M, Bonini L, Simone L, Rozzi S, Ferrari PF, Fogassi L (2012). Individual and social learning processes involved in the acquisition and generalization of tool use in macaques. Phil Trans R Soc B.

[CR27] Malmberg KJ, Xu J (2007). On the flexibility and the fallibility of associative memory. Mem Cognit.

[CR28] Manrod JD, Hartdegen R, Burghardt GM (2008). Rapid solving a problem apparatus by juvenile black-throated monitor lizards (*Varanus albigularis albigularis*). Anim Cogn.

[CR29] Millot S, Nilsson J, Fosseidengen JE, Bégout M-L, Fernö A, Braithwaite VA (2014). Innovative behaviour in fish: atlantic cod can learn to use an external tag to manipulate a self-feeder. Anim Cogn.

[CR30] Overington SE, Cauchard L, Côté K-A, Lefebvre L (2011). Innovative foraging behaviour in birds: what characterizes an innovator?. Behav Process.

[CR32] Papp S, Vincze E, Preiszner B, Liker A, Bókony V (2015). A comparison of problem-solving success between urban and rural house sparrows. Behav Ecol Sociobiol.

[CR33] Preiszner B, Papp S, Pipoly I, Seress G, Vincze E, Liker A, Bókony V (2017). Problem-solving performance and reproductive success of great tits in urban and forest habitats. Anim Cogn.

[CR34] R Core Team (2016) R: a language and environment for statistical computing. R Foundation for Statistical Computing, Vienna, Austria. https://www.R-project.org/

[CR35] Reader SM, Laland KN (2003). Animal innovation.

[CR36] Reichmuth Kastak C, Schusterman RJ (2002). Long-term memory for concepts in a California sea lion (*Zalophus californianus*). Anim Cogn.

[CR37] Sokal RR, Rohlf FJ (1995). Biometry: the principles and practice of statistics in biological research.

[CR38] St Clair JJH, Rutz C (2013). New Caledonian crows attend to multiple functional properties of complex tools. Phil Trans R Soc B.

[CR39] Taylor AH, Elliffe D, Hunt GR, Gray RD (2010). Complex cognition and behavioural innovation in New Caledonian crows. Proc R Soc Lond B.

[CR40] Thompson DC, Thompson PS (1980). Food habits and caching behavior of urban grey squirrels. Can J Zool.

[CR41] Thornton A, Samson J (2012). Innovative problem solving in wild meerkats. Anim Behav.

[CR42] van Horik JO, Madden JR (2016). A problem with problem solving: motivational traits, but not cognition, predict success on novel operant foraging tasks. Anim Behav.

[CR43] Wang M (2015) geesmv: Modified Variance Estimators for Generalized Estimating Equations. R package version 1.3. https://CRAN.R-project.org/package=geesmv

[CR44] Wang M, Long Q (2011). Modified robust variance estimator for generalized estimating equations with improved small-sample performance. Stat Med.

[CR45] Werdenich D, Huber L (2006). A case of quick problem solving in birds: string pulling in keas, *Nestor notabilis*. Anim Behav.

[CR46] Yan VX, Yu Y, Garcia MA, Bjork RA (2014). Why does guessing incorrectly enhance, rather than impair, retention?. Mem Cognit.

