# Supplementary material for: How to stay perfect: the role of memory and behavioural traits in an experienced problem and a similar problem
Source: Anim Cogn. 2017 Jul 11;20(5):941–52. doi: 10.1007/s10071-017-1113-7 (PMC5559565; doi:10.1007/s10071-017-1113-7)
Supplement: Supplementary file 1 — Supplementary material 1 (DOCX 17 kb) [file 10071_2017_1113_MOESM1_ESM.docx]

Supplementary materials for manuscript titled as ‘How to stay perfect: the role of memory and behavioural traits in an experienced problem and a similar problem.’

By Chow PKY, Lea SEG, Hempel de Ibarra N and Robert T

Table S1. Information about the five squirrels. Age for each squirrel is estimated from the time between their arrival at the laboratory and the current experiment, together with their status on arrival.

| Name | Sex | Estimated Age | Status | Participated experiments | | | | |
| --- | --- | --- | --- | --- | --- | --- | --- | --- |
|  |  |  |  | Social effect on cache decision  (K Jayne’s doctorate thesis^1^ or PKY Chow’s masters dissertation^2^) | Risk sensitivity^3^ | Simultaneous colour reversal learning on touch screen^4^ | Puzzle box^5^ | Serial reversal learning^6^ |
| Arnold | M | 11 | Rescued | ✓ | ✓ | ✓ | ✓ | ✓ |
| Leonard | M | 5 | Hand-raised | ✓ | ✓ | ✓ | ✓ | ✓ |
| Sarah | F | 5 | Hand-raised | ✓ | ✓ | ✓ | ✓ | ✓ |
| Simon | M | 9 | Rescued | ✓ | ✓ | ✓ | ✓ | ✓ |
| Suzy | F | 5 | Hand-raised | ✓ | ✗ | ✓ | ✓ | ✓ |

Note:

^1^ Jayne K (2014) Challenges faced by foraging Eastern Grey Squirrels, *Sciurus carolinensis*: competition, pilferage and predation risks. (Unpublished doctoral thesis). University of Exeter, Exeter, UK. https://ore.exeter.ac.uk/repository/handle/10871/15656

^2^ Chow PKY (2012) The effect of social condition on grey squirrels’ (*Sciurus carolinensis*) vigilant state and caching behaviour. (Unpublished masters dissertation). University of Exeter, Exeter, UK.

^3^No formal report available.

^4^Chow PKY, Leaver LA, Wang M, Lea SEG (2017) Touch screen assays of behavioural flexibility and error characteristics in Eastern grey squirrels (*Sciurus carolinensis*). Anim Cogn 20:459-471.

^5^ Chow PKY, Lea SEG, Leaver LA (2016) How practice makes perfect: the role of learning, flexibility, and persistence in problem solving efficiency. Anim Behav 112:273-283.

# ^6^ Chow PKY, Leaver LA, Wang M, Lea SEG (2015) Serial reversal learning in grey squirrels: learning efficiency as a function of learning and change of tactics. J Exp Psychol Anim Learn Cogn 41:343-353.
